# Factors associated with the intention to undergo Pap smear testing in the rural areas of Indonesia: a health belief model

**DOI:** 10.1186/s12978-021-01188-7

**Published:** 2021-06-30

**Authors:** Sumarmi Sumarmi, Yu-Yun Hsu, Ya-Min Cheng, Shu-Hsin Lee

**Affiliations:** 1 International Doctoral Program in Nursing, Department of Nursing, College of Medicine, National Cheng Kung University, Tainan, Taiwan; 2 Tanawali Persada School of Health Science, Takalar Regency, South Sulawesi, Indonesia; 3 Department of Nursing, College of Medicine, National Cheng Kung University, NCKU 1 University Road, Tainan, 70101 Taiwan; 4 Department of Obstetrics and Gynecology, College of Medicine, National Cheng Kung University, Tainan, Taiwan; 5 School of Nursing, Chung San Medical University, Taichung, Taiwan

**Keywords:** Rural, Health beliefs, Cervical cancer screening, Pap smear test

## Abstract

**Purposes:**

This study aimed to understand the influence of health beliefs, demographic factors, and health characteristics on the intention to undergo Pap smear testing among women in rural areas of Indonesia.

**Methods:**

A descriptive cross-sectional study was conducted and 687 married women participated in the study. A convenience sampling was applied to recruit the participants from community health centres in a rural region in Indonesia. Self-reported data using the Health Beliefs Model Scale for Cervical Cancer and Pap Smear Test was collected to assess the health beliefs. Independent t-tests, simple logistic regressions, and a hierarchical logistic regression with 3 steps were run. Statistical significance for analysis was set at *p* < 0.05.

**Results:**

The mean age of the participants was 42 years (SD = 8.4). Among the participants, 81% of the women had never undergone a Pap smear test, and 61% (n = 422) of the women reported a high intention of receiving a Pap smear test. Income and education Health beliefs regarding Pap smear testing were different between women who had low and high intentions to undergo Pap smear testing. Health beliefs, such as perceived benefits, severity, barriers to Pap smear testing, and health motivation for a Pap smear test were associated with the intention to undergo Pap smear testing among rural Indonesian women. Overall, the hierarchical multiple regression with 3 steps containing demographic, health characteristics, and health belief variables accounted for 31% variance of the intention to undergo Pap smear test among the Indonesian rural women.

**Conclusions:**

Low screening rates of cervical cancer and high intentions to do the screening exist among rural Indonesian women. Health beliefs significantly affect the rural women’s intention of Pap smear testing in Indonesia.

## Plain language summary

Cervical cancer is a leading cancer among women and a significant cause of mortality for females around the world, including Indonesia. Globally, the screening rate for cervical cancer among women in rural areas remains low. In Indonesia, the incidence and the mortality from cervical cancer remain high compared to other female cancers. The Indonesian government has offered a free Pap smear screening to women since 2014, but the screening rate is still low, around 28%.

A total of 687 married women were included in the study. Approximately 80% of Indonesian women living in rural areas have never undergone a Pap smear test, and 60% of women reported a high intention of receiving a Pap smear test. Education, income, previous experience of Pap smear testing, a friend with a history of cervical cancer, perceived severity, perceived benefits, perceived barriers, and health motivations were significantly associated with the intention of Pap smear testing. Low screening rates of cervical cancer and high intentions toward the cervical cancer screening exist among rural Indonesian women. Health beliefs significantly affect the women’s intention of Pap smear testing.

## Introduction

The incidence of cervical cancer in developing countries is disproportionately high (> 80%) [1]. According to World Health Organization (WHO) data, in 2018 cervical cancer was the second leading cause of female cancer deaths in Indonesia, with an incidence of 20.928 cases and mortality of 9.498 cases per 100,000 women.

In Indonesia, approximately 70% of women with cervical cancer are in the advanced stage of the disease when they visit hospitals for initial treatment. At this point, the cervical cancer cells have already infiltrated the parametrium [2]. Pap smear test has been recommended for detecting cervical cancer at early stage which is highly treatable [3]. The Indonesian government has been offering free Pap smear tests for married women aged 30 years and above since 2014, whereas a visual Inspection with Acetic Acid (VIA) has widely used for several decades as an initial step in cervical cancer screening for Indonesian women [4].

Prior studies revealed that socio-cultural and economic conditions affected the receptiveness of cervical cancer screening among rural women in Indonesia. Women with low education and income levels faced obstacles in obtaining sufficient information about cervical cancer and cervical cancer screening [5]. Implementation of cervical cancer screening in rural areas is extremely challenging due to limited availability, affordability, and accessibility of cervical cancer screening services for women who are socio-economically disadvantaged [6, 7].

Intention means someone is ready to engage in a specific behaviour or the thought to perform the behaviour [8]. The intention to undergo cervical cancer screening refers to women who are willing to be engaged in cervical cancer screening. Research has found that women who have a higher intention to receive a Pap smear test will more regularly get Pap smear testing [9]. Studies from rural Thai, Nigerian and North Korean women revealed that women’s participation in cervical cancer screening was affected by demographic and health belief factors such as administrative failures, the unavailability of a female screener, fear of embarrassment, lack of health insurance, limited access to health care services, lack of clinician recommendations, acculturation, and lack of awareness [10–12].

The Health Beliefs Model (HBM) focuses on the determinants of health-related behaviours, with the determinants consisting of perceived susceptibility and severity of a health problem, perceived benefits and barriers of conducting health-related behaviours, cues to action, and other sociodemographic factors [13]. According to the HBM, women are more likely to engage in health seeking behaviours if their perceptions of susceptibility and seriousness with regard to a specific health condition are high, the barriers to carrying out such behaviours are low, and the benefits of engaging in health behaviours are significant [14]. Although the relationship between the intention to undergo Pap smear testing and the health beliefs of Pap smear testing has been investigated in women living in non-western societies [15–18], until now, no research specific to rural Indonesian women’s intentions and health beliefs regarding cervical cancer screening has yet been conducted. In this study, cervical cancer screening referred to Pap smear testing. The primary purpose of this study was to investigate the relationship between intentions and health beliefs regarding Pap smear testing among Indonesia women in rural areas. Second, the study examined the predictors of demographic and health related factors on the intention to undergo Pap smear testing.

## Methods

### Study design

A descriptive cross-sectional design was conducted in the study with a convenience sampling.

### Setting and participants

Participants were recruited from 15 community health centres in Takalar District. Takalar District is a rural area on Sulawesi Island, which is located 30 km from the capital of South Sulawesi Province, Indonesia. Based on the 2018 Central Statistical Agency, Takalar District had 295,892 inhabitants, with 51.9% of the population being women [19]. The study participants were women aged ≥ 30 years old, married, and able to communicate using Bahasa Indonesia regardless of literacy status. Women with a history of cervical cancer or hysterectomy were excluded from the study because the USPSTF recommends against Pap smear screening among patients who have had a hysterectomy and cervical cancer [3]. A minimum sample size of 553 was determined using G*Power software version 3.1.9.2 to carry out the logistic regression with the assumption of α = 0.05, power level = 0.8, and an odds ratio of 1.35 as a reference from a pilot study of the research.

### Measurements

The Health Beliefs Model Scale for Cervical Cancer and Pap Smear Test (HBMSCCPS) was used to assess health beliefs regarding Pap smear tests [20]. The HBMSCCPS consists of 35 items which are divided into five subscales: susceptibility to cervical cancer, perceived severity of cervical cancer, benefits of Pap smear tests, barriers to Pap smear tests, and health motivation. Each item is answered using a five-point Likert scale from strongly disagree (scores 1) to strongly agree (scores 5). The higher score represents positive beliefs and attitudes related to Pap smear screening behaviour, except for the subscale of barriers which have a negative association. The test–retest reliability of the HBMSCCPS ranged from 0.79 to 0.87 and the Cronbach’s alphas in the five subscales ranged from 0.62 to 0.86 in a study which examined the health beliefs of cervical cancer screening among Turkish women [20].

Permission to translate the HBMSCCPS into the Indonesian language was obtained from the scale’s developer. A cross-cultural adaptation with a forward and backward process was applied to the scale translation [21]. After translation, the scale was evaluated by a panel committee consisting of six experts in women’s health. The item-level content validity index (I-CVI) was 0.99 and the scale-level content validity index (S-CVI) was 0.97. Internal consistency of the HBMSCCPS was satisfactory in the study, with Cronbach’s alpha for the five subscales ranging from 0.73 to 0.92 (susceptibility = 0.82, severity = 0.78, benefits = 0.92, barriers = 0.87 and health motivation was 0.73).

Intention to undergo cervical cancer screening was assessed by asking participants the one question: “What is your likelihood of performing cervical cancer screening?” This item was responded to using an 11-point Likert scale from 0 (no intention) to 10 (strongest intention). The item of intention was then classified as “low” or “high” intention by the median score of the intention item as a cut-off point. A median score of health behaviour intention has been applied in one study which examined the HPV vaccination intention [22]. A single-item measure has been used to assess the perceived probability of behaviour performance under the expectancy theory [23] and work-related well-being [24].

The demographic and health characteristics were obtained from participants using a self-reported demographic and health characteristics sheet. The demographic information included age (years), marital status (married/widowed), level of education (low/middle/high), ethnicity (Makassar/non-Makassar), occupation (unemployed/employed), income level based on Indonesian wage (no//low/high), religion (Muslim/Christian). Furthermore, the health characteristics included parity (yes/no), awareness of cervical cancer (yes/no), awareness of Pap smear testing (yes/no), previous experience of cervical cancer screening (regular, irregular and never), family history of cervical cancer (yes/no), and friend’s history of cervical cancer (yes/no).

### Study procedure

Ethical approval was obtained from the Institutional Review Board of the Faculty of Medicine at Muhammadiyah Jogyakarta University (No.004/EP-FKIK-UMY/X/2016). Additionally, permission to conduct the research was acquired from the heads of the community health centres prior to conducting the study. A convenience sampling was applied for recruiting participants. The health care providers and staff looked for potential participants among women visiting the community health centres, and then referred potential participants to the researchers, including the principal investigator and five research assistants. If women met the inclusion criteria and agreed to participate in the study, they were asked to complete a written consent and fill out questionnaires, including the HBMSCCPS, intention to undergo Pap smear testing, and the Demographic and Health Characteristics sheet. The investigators checked the completeness of the questionnaires after the participants filled out them. Data were collected from August to October 2016. The study recruited 687 women, and 682 women completed the questionnaires.

### Data analysis

The data were analysed using SPSS version 17. Descriptive statistics (frequencies, percentages, mean and standard deviation) were used to present demographic factors and health characteristics. Independent t-tests were applied to compare the health beliefs between the women with high and low intention to undergo Pap smear testing. Dummy variables were used for the categorical variables: education level (low, middle, and high), income level (no, low and high), and previous experience of Pap smears test (regular, irregular and never).

Simple logistic regression was used to identify significant individual variable (i.e., demographic factors, health characteristics, and the five domains of health belief for the intention of Pap smear testing. After that, the significant variables were entered into a hierarchical logistic regression with three steps to build a model. The orderings of the three-step were as follows: demographic variables, health characteristics, and five domains of health belief. The order of the entry variables was set up based on the theory of the health belief model. Variance inflation factors (VIF) were assessed to examine whether multicollinearity existed between the explanatory variables, and the results showed no evidence of high collinearity (maximum VIF = 2.32, Minimum VIF = 1.04, Mean VIF = 1.44).

## Results

### Demographic factors and health characteristics

The mean age of the 685 women was 42.3 years (SD = 8.4; range 30–74). Ninety-two percent of the women were married, 8.1% of participants being widowed, and 91% of them having children (*n* = 623). In terms of education level, 52% of participants (*n* = 359) were graduated from junior or senior high school. More than half (54%) of respondents (*n* = 369) were unemployed and had no income. Most of the participants were Muslim (*n* = 672, 98%).

The majority of participants (*n* = 554, 81%) had never undergone Pap smear testing. More than one quarter (*n* = 192, 28%) had never heard of cervical cancer and 33% (*n* = 227) had never heard of the Pap smear test. A small percentage (*n* = 36, 5%) had a family history of cervical cancer, and only 6% (*n* = 42) reported having friends with a history of cervical cancer (Table 1).

**Table 1:** Demographic factors and health characteristics of study sample (*n* = 682)

Characteristic	Mean (*n)*	SD (%)
Age (years)	42.3	8.4 (30–74)
Marital status		
Married	627	91.9
Widowed/divorced	55	8.1
Level of education		
Low education (≤ Elementary school)	198	29.0
Middle education (Junior and high school)	359	52.6
High education (≥ College)	125	18.3
Ethnicity		
Makassar	592	86.8
Non-Makassar	90	13.2
Occupation		
Unemployed	369	54.1
Employed	313	45.9
Income level		
No income	369	54.1
Low income (< $200 US)	168	24.6
High income (≥ $200 US)	145	21.3
Religion		
Muslim	672	98.5
Christian	10	1.5
Parity		
No children	59	8.7
Have children	623	91.3
Awareness of cervical cancer		
Yes	490	71.8
No	192	28.2
Awareness of Pap smear testing		
Yes	455	66.7
No	227	33.3
Previous experience of Pap Smear test		
Regular (every 1–3 years)	33	4.8
Irregular	95	13.9
Never	554	81.2
Family history of cervical cancer		
Yes	36	5.3
No	646	94.7
Friends’ history of cervical cancer		
Yes	42	6.2
No	640	93.8

### Health beliefs and the intention to undergo Pap smear testing

The median score of cervical cancer screening intention was 7.00 (mean = 6.9), which was used as the cut-off point to categorize low (0–6) and high (7–10) intention to undergo Pap smear testing. A total of 61% of the participants (*n* = 422) reported a high intention and 39% of the participants (*n* = 260) reported a low intention to undergo Pap smear testing. The comparisons between high and low intention to undergo Pap smear testing were performed from the total scale and each subscale of HBMSCCPS, respectively. The independent t-tests showed that, between women with high intention and low intention to undergo Pap smear testing, there were significant differences in the four subscales of HBMSCCPS, namely perceived severity (*t* = -3.37, *p* =  < 0.01), perceived benefits (*t* = -4.51, *p* =  < 0.001), perceived barriers (*t* = 3.92, *p* =  < 0.001), and health motivation (*t* = -5.05, *p* =  < 0.001). The women who had high intention of undergoing Pap smear testing had higher perceived severity, benefits, health motivation, and lower barriers regarding Pap smear testing than those women who had low intention of undergoing Pap smear testing. However, both groups had a similar total score of the HBMSCCPS and subscale of perceived susceptibility (Table 2).

**Table 2:** Relationships between health beliefs and the intention to undergo cervical cancer screening (*n* = 682)

Variable	Intention to undergo cervical cancer screening		
	Low intention (*n* = 260)	High intention (*n* = 422)	*t*
	Mean ± SD	Mean ± SD	
Health beliefs	109.61 ± 13.25	110.82 ± 17.01	− 1.03
Perceived susceptibility	6.84 ± 2.37	6.54 ± 2.75	1.50
Perceived severity	20.35 ± 4.68	21.67 ± 5.45	− 3.37**
Perceived benefits	30.89 ± 6.48	33.22 ± 6.58	− 4.51***
Perceived barriers	39.29 ± 9.03	36.46 ± 9.27	3.92***
Health motivation	12.24 ± 1.74	12.93 ± 1.72	− 5.05***

*t*: Independent *T*-test

*p < 0.05; **p < 0.01; ***p < 0.001

### Independent variables of the intention to undergo Pap Smear testing

Table 3 reveals the results of the simple logistic regression showing five demographic factors significantly associated with the intention to undergo Pap smear testing: marital status (OR: 2.07, 95% CI: 1.19–3.61), low education level (OR: 0.63, 95% CI: 0.41–0.94), high education level (OR: 3.58, 95% CI: 2.59–4.95), low-income level (OR: 0.50, 95% CI: 0.35–0.72) and high-income level (OR: 2.48, 95% CI: 1.63–3.80). Married women with a high education level and high income were more likely to have high intention to undergo Pap smear testing. In contrast, women who had low income and a lower education level were likely to have a low intention of undergoing Pap smear testing.

**Table 3:** Independent variables of the intention to undergo cervical cancer screening (*n* = 682)

Variable included	Intention to undergo cervical cancer screening				
	B	SE	Wald	OR	CI (95%)
Demographic factors					
Age	− 0.01	0.01	1.88	0.99	0.97–1.00
Marital status					
Married	0.73	0.28	6.61	2.07*	1.19–3.61
Widowed/divorced				1.0	
Level of education^a^					
Low education	− 0.47	0.21	5.00	0.63*	0.41–0.94
Middle education				1.0	
High education	1.27	0.17	59.35	3.58***	2.59–4.95
Ethnicity					
Makassar	− 0.36	0.24	2.14	0.70	0.43–1.13
Non-Makassar				1.0	
Occupation					
Employed	0.03	0.16	0.04	1.03	0.76–1.41
Unemployed				1.0	
Income level					
No income				1.0	
Low income (< $200 US)	− 0.68	0.18	14.45	0.50***	0.35–0.72
High income (≥ $200 US)	0.91	0.22	17.68	2.48***	1.63–3.80
Religion					
Muslim	− 0.91	0.79	1.32	0.40	0.08–1.90
Christian				1.0	
Parity					
No children				1.0	
Have children	0.12	0.28	0.18	1.12	0.65–1.94
Health characteristics					
Awareness of cervical cancer (Ref: no)	1.36	0.18	58.23	3.91***	2.75–5.55
Awareness of Pap smear test (Ref: no)					
Previous experience of test	0.95	0.17	32.41	2.59***	1.87–3.60
Regular	2.32	0.73	10.04	10.23**	2.43–43.10
Irregular				1.0	
Never	− 1.29	0.25	26.69	0.28***	0.17–0.45
Family history of cervical cancer (Ref: no)	1.66	0.54	9.56	5.25**	1.83–15.03
Friends’ history of cervical cancer (Ref: no)	2.16	0.60	12.84	8.72***	2.67–28.53
Health beliefs domain					
Perceived susceptibility	− 0.04	0.03	2.09	0.96	0.90–1.02
Perceived severity	0.05	0.01	10.29	1.05**	1.02–1.08
Perceived benefits	0.05	0.01	18.75	1.05***	1.03–1.08
Perceived barriers	− 0.03	0.01	14.71	0.97***	0.95–0.98
Health motivation	0.23	0.05	23.69	1.25***	1.14–1.37

*SE* standard error, *OR* odds ratio, *CI* confidence interval

^a^Low Education: ≤ Elementary school); Middle Education: Junior and high school; High Education: ≥ College

*p < 0.05; **p < 0.01; ***p < 0.001

In addition, six health-related factors associated with the intention to undergo Pap smear testing were (Table 3): awareness of cervical cancer (OR: 3.91, 95% CI: 2.75–5.55), awareness of Pap smear testing (OR: 2.59, 95% CI: 1.87–3.60), had regular Pap smear tests (OR: 10.23, 95% CI: 2.43–43.10), never had a Pap smear test (OR: 0.28, 95% CI: 0.17–0.45), family history of cervical cancer (OR: 5.25, 95% CI: 1.83–15.03), and friend’s history of cervical cancer (OR: 8.72, 95% CI: 2.67–28.53). Women who had regular Pap smear tests, family or friends with a history of cervical cancer, awareness of cervical cancer, or awareness of Pap smear testing were more likely to have high intention to undergo Pap smear testing.

Four variables of the health beliefs significantly associated with the intention to undergo Pap smear testing were (Table 3): perceived severity (OR: 1.05, 95% CI: 1.02–1.08), perceived benefits (OR: 1.05, 95% CI: 1.03–1.08), perceived barriers (OR: 0.97, 95% CI: 0.95–0.98), and health motivation (OR: 1.25, 95% CI: 1.14–1.37). These results revealed that the women who considered cervical cancer as a severe disease, perceived the benefits of screening action, had fewer barriers to undergoing screening, and had high motivation were more likely to have high intention to undergo Pap smear testing.

### Final Logistic Regression Model of the Intention to undergo Pap Smear Testing.

Hierarchical multiple logistic regression with three steps was run to identify the model of intention to undergoing Pap smear testing as an outcome variable (Table 4). In Step 1, the significant variables were: high level of education (B = -1.59, *p* =  < 0.001), low level of income (B = 0.98, *p* =  < 0.001). The demographic variables in the step 1 accounted for 17% of the variance (Nagelkerke R^2^) of intention of Pap smear test. In step 2, after controlling for the demographic variables, the significant health characteristics variables related to undergoing Pap smear testing included awareness of cervical cancer (B = -1.07, *p* =  < 0.001), awareness of Pap smear testing (B = 0.45, *p* =  < 0.05), never done Pap smear test (B = 1.02, *p* =  < 0.01), friend with a history of cervical cancer (B = -1.56, *p* =  < 0.05). In Step 3, after controlling for the demographic variables and health characteristics, the significant health belief variables related to undergoing Pap smear testing included perceived severity of screening (B = 0.05, *p* =  < 0.05), perceived benefits of screening (B = 0.04, *p* =  < 0.01), perceived barriers of screening (B = -0.02, *p* =  < 0.05), and health motivation (B = 0.11, *p* =  < 0.05). Table 4 illustrates the variance increased from 26% at the Step 2 to 31% at the Step 3. It indicates that the five health belief variables in Step 3 only explained a small segment variance of the intention of undergoing Pap smear test (R^2^ = 0.05).

**Table 4:** Final logistic regression of the intention to undergo cervical cancer screening (*n* = 682)

Variable included	Model 1				Model 2				Model 3			
	B	SE	OR	CI (95%)	B	SE	OR	CI (95%)	B	SE	OR	CI (95%)
Marital status (Ref: Widowed/divorced)	− 0.49	0.31	0.61	0.34–1.12	− 0.39	0.32	0.67	0.36–1.26	− 0.39	0.33	0.68	0.36–1.29
High education (Ref: Middle education)	− 1.59	0.22	0.20***	0.13–0.32	− 1.11	0.25	0.33***	0.20–0.54	− 1.00	0.26	0.37***	0.22–0.61
Low education (Ref: Middle education)	− 0.43	0.25	0.65	0.40–1.06	− 0.16	0.27	0.85	0.50–1.43	− 0.07	0.27	0.93	0.55–1.60
High income (Ref: No income)	0.18	0.27	12.1	0.71–2.05	0.51	0.29	1.67	0.95–2.93	0.59	0.29	1.81*	1.02–3.22
Low income (Ref: No income)	0.98	0.21	2.67***	1.75–4.08	1.02	0.22	2.78***	1.79–4.32	1.00	0.23	2.72***	1.72–4.30
Awareness of cervical cancer (Ref: no)					− 1.07	0.28	0.34***	0.20–0.59	− 1.02	0.29	0.36***	0.20–0.63
Awareness of Pap smear testing (Ref: no)					0.45	0.27	1.58*	0.93–2.67	0.57	0.28	1.77*	1.03–3.06
Regularly had experienced of Pap smear test					− 0.74	0.80	0.48	0.10–2.29	− 0.87	0.83	0.42	0.08–2.13
(Ref: Irregular Pap smear test)												
Never done Pap smear test					1.02	0.31	2.79**	1.53–5.08	1.16	0.32	3.19***	1.69–6.02
(Ref: Irregular Pap smear test)												
Family history of cervical cancer (Ref: no)					− 0.88	0.57	0.41	0.13–1.28	− 0.88	0.59	0.41	0.13–1.31
Friends’ history of cervical cancer (Ref: no)					− 1.56	0.64	0.21*	0.06–0.74	− 1.49	0.66	0.22*	0.06–0.81
Perceived severity									0.05	0.02	1.05*	1.01–1.09
Perceived benefits									0.04	0.01	1.04**	1.01–1.07
Perceived barriers									− 0.02	0.01	0.97*	0.95–1.00
Health motivation									0.11	0.06	1.12*	1.00–1.25
R^2^	0.17				0.26				0.31			
ΔR^2^					0.09				0.05			

*SE* standard error, *OR* odds ratio, *CI* confidence interval

*p < 0.05; **p < 0.01; ***p < 0.001

## Discussion

This study was performed to identify the relationship between rural Indonesian women’s health beliefs and intention to undergo Pap smear testing. The findings of this study indicated that, in general, among rural Indonesian women, 61% had high intention to undergo Pap smear testing, however only 19% of participants had done the test. On the other hand, 39% of the rural Indonesia women had low intention to undergo Pap smear testing and Sociodemographic background (i.e., education level, income), family history of cervical cancer, as well as awareness of cervical cancer and cervical cancer screening were associated with the intention of undergoing Pap smear testing. Furthermore, women have a high intention of undergoing Pap smear testing if they perceive cervical cancer as a severe disease, take action to prevent cervical cancer, reduce the barriers to Pap smear testing, and have high motivation.

Indonesian national health insurance has fully covered Pap smear testing for married women over 30 years of age since 2014, nonetheless, 81% of the participants have never undergone a Pap smear test, 28% of the women had never heard of cervical cancer, and 33% had never heard of the Pap smear test. However, the awareness of cervical cancer or Pap smear tests were related to intention of undergoing Pap smear testing. Higher intention to have Pap smear testing was found in women who had heard of cervical cancer or Pap smear testing, while lower intention was the case among those who had not. These findings were consistent with a previous study among Omani women and nurses in Ghana which stated that limited information and knowledge of cervical cancer and Pap smear testing contributed to low participation in cervical cancer screening [25–27]. This study provided data that one third of the women only had an elementary level education. This low level of education may limit the women’s health literacy regarding cervical cancer and cervical cancer screening. Health education related cervical cancer screening has been considered as an effective strategy to improve the participation rate of cervical cancer [28–30]. Future study is needed to tailor an appropriate educational program to improve women’s literacy of cervical cancer and Pap smear tests among women in rural Indonesian areas.

The current study found that income is significantly associated with the intention of undergoing Pap smear testing. In the present study, the low-income women have low intention toward taking Pap smear test, whereas and the high-income women have high intention toward taking Pap smear test. Similar findings have been reported from studies in Thailand, which found that Thai women who had high monthly income were more likely to have a Pap smear test [5, 10]. A majority of the women in this study were low income or no income, low education, and unemployed. Although Pap smear testing is generally free of charge in Indonesia, the low-income women living in rural areas might be worried about losing hourly wages when leaving their jobs to undergo Pap smear testing. Future research is needed to explore the financial and social issues regarding cancer screening among low-income women.

Our study reported higher perceived severity and perceived benefits were associated with a higher intention to undergo Pap smear testing. This result was consistent with the previous study in which Vietnamese Americans women believed that they were at higher risk of getting cervical cancer than average; those women believed that Pap smear tests were the best method to detect cervical cancer, and the disease of cervical cancer is easily treated if detected early [31, 32]. Women might have an increased motivation to undergo cervical cancer screening if they are aware of cervical cancer as a threat. The majority of women in this study had never had a Pap smear test, but had a high intention toward receiving a Pap smear test. It may indicate a motivation is a force to initiate a behaviour. Motivation may arise from the awareness of cervical cancer and the benefit of early detection by Pap smear testing [33]. Both studies in Turkey and in China had reported that women who had undergone a Pap smear test had higher health motivation [34, 35].

Our study shows low perceived barriers were associated with high intention to undergo Pap smear testing. The obstacles of undergoing Pap smear test include poor accessibility of health care service, fear of painful procedures, fear of bad results, unknowing where to go for a Pap smear test, reluctance to show the private body arts during examinations, unavailability of a female screener (physician), and being unable to afford the test. Similar findings are found from Iran, Thailand and Korean American women that proposed the above barrier factors hindering their action to cervical cancer screening [13, 36, 37]. Healthcare providers should recognize these barriers and discuss them with women who are eligible to receive a free Pap smear test. It is recommended to set up private, comfortable, and women-friendly examination environment for cervical cancer screening.

The final multivariate model accounted for 31% of the variance of intention to undergo Pap smear testing. The variance in the current study is higher than that in one previous study from Singapore, which only explains 20.4% variance [38]. However, the final block containing health belief variables only explained 5% of the variance of intention to undergo Pap smear testing. It is possible that other factors contribute to the intention of undergoing Pap smear testing, such as knowledge, accessibility to health services, and cues to action. Future studies are needed to explore the above factors and whether they are related to the intention or behaviour of Pap smear testing.

## Limitations

The present study has several limitations. Firstly, data were collected from women living in Takalar Regency, which is a rural area, and the homogeneity of the sample population may limit the findings’ generalization to women living in urban areas of Indonesia. Secondly, this study used a convenience sample and cross-sectional study design, which cannot assume the variables were causally related. Selection bias may come from the convenience sample, which leads to recall or social desirability bias. Thirdly, the study does result of this study can only be generalized to population with similar characteristics. This study recruited only married women over 30 years of age as its subjects, thus the results cannot be generalized to unmarried women, or women of a younger age. To deal with these limitations, future studies are suggested to expand the geographical location to both rural and urban areas, as well as a longitudinal study designed and recruit both single and married women, to explore their intention of undergoing Pap smear testing and related health beliefs. Finally, the study used a single item to measure the intention of undergoing a Pap smear test among rural women. We cannot exclude the low reliability of the single-item scale. Future studies should examine the reliability of the single-item intention of undergoing a Pap smear test.

### Implications for practice and/or policy

Women’s health beliefs influence their intention of undergoing Pap smear testing. Better understanding in women’s awareness and knowledge regarding cervical cancer and Pap smear testing is important in developing countries. It is necessary to initiate a campaign emphasizing the benefits of early detection of cervical cancer and providing detailed information about the Pap smear testing to improve the screening motivation for Indonesian adult women. Information about cervical cancer and its screening should be organized and implemented throughout public media systems, such as TV, broadcast, internet, and newspaper. Even though Pap smear testing services are free of charge to qualified Indonesian married women, financial issues such as transportation fee may still be an obstacle among low-income and unemployed women. A transportation subsidy or assistance for low-income women to travel to health community centres could be a strategy for promoting screening rates in rural areas. Furthermore, providing accessible resources such as mobile cervical cancer screening services might become an additional solution in improving cervical cancer screening test coverage for women living in rural areas.

## Conclusions

The health beliefs such as perceived benefits and health motivation for a Pap smear test are associated with the intention to undergo Pap smear testing among Indonesian women living in rural areas. Demographic factors such as income and education, together with health-related factors such as awareness, previous screening experience, and a friend’s history of cervical cancer are the significant variables associated with women’s intention of undergoing Pap smear testing. Health care professionals should understand barriers regarding Pap smear testing for women with low intention to undergoing screening. An educational program of cervical cancer screening should incorporate cultural information and communication skills. Health care professionals should understand that women with low education and income are a vulnerable population, having low intention to receiving Pap smear testing.

## Data Availability

Availability of data and material are available upon request from the corresponding author.

## Abbreviations

HBM: Health Beliefs Model (HBM)
I-CVI: Item-level Content Validity Index
S-CVI: Scale-level Content Validity Index
HBMSCCPS: The Health Beliefs Model Scale for Cervical Cancer and Pap Smear Test
VIA: Visual Inspection with Acetic Acid
WHO: World Health Organization
